# Early Rehabilitation Versus Conventional Approaches in Post-Traumatic Hand Injuries with Multiple Lesions: Clinical Outcomes and Future Directions

**DOI:** 10.3390/medicina61112063

**Published:** 2025-11-19

**Authors:** Adriana Serban, Andreea Grosu-Bularda, Eliza-Maria Bordeanu-Diaconescu, Georgiana-Ozana Tache, Marius Stoica

**Affiliations:** 1 Institute of Doctoral and Postdoctoral Studies, National University of Physical Education and Sports, 060057 Bucharest, Romania; 2 Clinical Emergency Hospital of Bucharest, 014461 Bucharest, Romania; 3 Department 11, Discipline Plastic and Reconstructive Surgery, University of Medicine and Pharmacy Carol Davila, 050474 Bucharest, Romania; 4 Department 9, Physical Medicine and Rehabilitation, University of Medicine and Pharmacy Carol Davila, 050474 Bucharest, Romania

**Keywords:** early rehabilitation, hand trauma, hand injuries, rehabilitation program, delayed rehabilitation

## Abstract

*Background and Objectives:* Complex hand injuries often lead to long-term functional impairment and require structured rehabilitation following surgery. While early rehabilitation may improve outcomes by preventing stiffness and adhesions, it can also increase pain and psychological distress. In contrast, delayed rehabilitation may offer short-term comfort but risks slower recovery. The study aimed to compare the outcomes of early versus delayed rehabilitation through a four-phase therapeutic protocol, with standardized assessments at baseline, 4 weeks, and 12 weeks. *Materials and Methods*: This study included 90 patients with complex hand trauma who underwent emergency surgical intervention followed by a structured rehabilitation program. Key parameters included active range of motion (TAM), grip strength, pain (VAS), edema, hand function (QuickDASH), and anxiety levels (GAD-7). Statistical analysis was used to evaluate differences in physical and psychological recovery over time between the two rehabilitation approaches. *Results:* This study demonstrated that both early and delayed postoperative rehabilitation significantly improved physical and psychological outcomes in patients with complex hand trauma. However, early rehabilitation was associated with faster resolution of edema, quicker gains in functional mobility, and earlier improvement in grip strength, despite causing higher initial levels of pain and anxiety. Conversely, delayed rehabilitation resulted in lower early pain and anxiety but showed slower functional recovery. Subgroup analysis revealed that patients with flexor tendon injuries benefited most from early rehabilitation in terms of mobility, strength, and anxiety reduction, while those with multifocal or complex injuries achieved greater long-term pain relief. *Conclusions:* As rehabilitation continues to evolve, the adoption of personalized, multimodal, and technologically integrated strategies holds promise for improving both the speed and quality of recovery while addressing the psychological and functional dimensions of patient care. Overall, the study supports early, structured, and individualized rehabilitation protocols, emphasizing a multidisciplinary approach that integrates both physical and psychological recovery strategies.

## 1. Introduction

Hand injuries represent one of the most frequent and functionally disabling categories of musculoskeletal trauma, accounting for approximately 6–28% of all such cases presenting to emergency departments [1,2]. These injuries are often complex, simultaneously affecting skeletal structures, tendons, nerves, vessels and surrounding soft tissues. Therefore, they frequently require surgical management followed by prolonged and sustained rehabilitation. The consequences of such injuries include joint stiffness, reduced grip strength, impaired sensibility and persistent pain. These cause significant limitations in activities of daily living with long-term implications for occupational reintegration and quality of life [3,4].

Rehabilitation plays a significant role in functional restoration, yet there is no universal consensus on the optimal timing and structure of interventions. Early initiation of rehabilitation has been advocated to prevent adhesions, joint stiffness and secondary contractures, thereby facilitating tendon gliding and supporting superior long-term outcomes [2,5]. However, the beginning of therapy immediately after surgery may also increase pain, edema, and psychological distress, factors that could hinder adherence and compromise recovery. Conversely, delayed rehabilitation, typically started after a period of immobilization, is associated with greater short-term comfort but risks long-term rigidity, reduced tendon excursion and slower functional progress [6].

Most published studies have examined isolated injuries, particularly flexor tendon repairs, whereas patients with multifocal or combined lesions, such as fractures, peripheral nerve injuries or vascular compromise, have been less frequently studied. This represents a gap in the literature, as functional recovery trajectories may vary significantly depending on injury type and complexity [6]. Furthermore, few investigations have systematically addressed the interaction between physical recovery and psychological outcomes, even though anxiety, fear of movement and maladaptive pain responses are known to affect rehabilitation success [7,8].

Alongside classical physiotherapeutic modalities such as manual mobilization, scar management, and strengthening and physical modalities such as ultrasound and electrotherapy, recent years have seen the emergence of multimodal strategies designed to enhance recovery. These include mirror therapy, graded motor imagery, desensitization techniques and customized orthotic positioning. Evidence suggests that these approaches may stimulate cortical reorganization, reduce pain, and support motor relearning [9,10,11,12,13].

However, their systematic integration into structured, stage-based rehabilitation protocols for complex hand trauma has been insufficiently documented in clinical practice.

Given these gaps, the study aimed to compare the impact of early versus delayed initiation of rehabilitation therapy in patients with complex hand trauma. Specifically, the study aimed to assess the differences in functional recovery, pain control, muscle strength, edema reduction and psychological status between the two rehabilitation approaches.

## 2. Materials and Methods

### 2.1. Study Design and Population

A retrospective study was conducted at the Emergency Clinical Hospital of Bucharest in Romania over a period of 2 years, from July 2023 to July 2025. A total of 90 patients diagnosed with complex hand trauma were included in the study, all of whom underwent emergency surgical intervention and followed a rehabilitation program afterward. Patients were eligible for inclusion if they presented with injuries located distal to the carpal bones and had provided informed written consent. Exclusion criteria included patients with decompensated systemic diseases, isolated injuries involving only a single tendon or a single bone fracture, infections, arthritis, or central motor neuron involvement. The study was conducted retrospectively, on anonymized patient data collected during a doctoral research project, with appropriate ethical approval. All data were collected from existing medical records of patients who had received treatment according to the same structured therapeutic protocol, and no interventions were prospectively assigned or modified for research purposes.

### 2.2. Therapeutic Protocol

**Figure 1:**
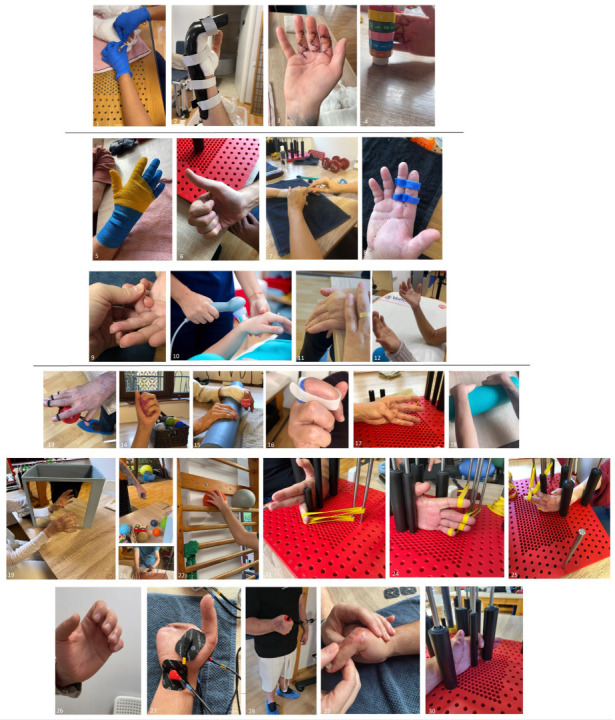
The therapeutic program (images 1–4 depict the Immediate postoperative phase; images 5–12 depict the Early rehabilitation phase; images 13–30 depict the Intermediate phase and the Advanced functional reintegration phase). 1-passive mobilizations, 2-customized orthosis, 3,4-early tendon gliding movements, 5-compressive bandage, 6,12-tendon gliding movements, 7,9-passive mobilizations, 8-customized orthosis, 10-ultrasound therapy, 11-passive-active mobilization, 13,14,15-strengthening exercises, 16-customized orthosis, 17-active ROM, 18-passive-active ROM, 19-mirror therapy, 20-grip strength, 21-prono-supination ROM and grip strength, 22-proprioception, 23,24,25,30-ROM increase postures, 26-tendon gliding, 27-electrotherapy, 28-strengthening exercises, 29-passive and passive-active mobilizations.

The therapeutic program was structured into four progressive phases, each defined by specific objectives and tailored interventions corresponding to the biological healing stage, as depicted in Figure 1, which consisted of the following:

- Immediate postoperative phase (0–14 days): This phase was only applicable to patients who underwent early rehabilitation, initiated shortly after surgical intervention. The priority during the acute phase was to control inflammation, limit edema, protect sutured tissues and prevent complications such as infection, secondary tissue damage or adhesions. Functional goals also included pain reduction and maintenance of mobility in uninvolved joints (elbow, shoulder, cervical spine) to avoid secondary stiffness. Interventions included lymphatic drainage massage (to promote venous and lymphatic return), cryotherapy for edema and pain control, passive and passive-assisted mobilization to maintain joint congruency and early tendon-gliding movements aimed at minimizing adhesions. Customized protective orthoses were used: dorsal blocking splints for flexor tendon repairs, volar blocking splints for extensor injuries and individualized “intrinsic plus” positioning to maintain optimal joint alignment and ligament tension.
- Early rehabilitation phase (2–6 weeks): This phase aimed at preventing stiffness and contractures while allowing controlled mobilization of healing tissues. Techniques focused on maintaining the flexibility of periarticular structures, promoting safe tendon excursion and gradually initiating functional use of the hand. Scar massage and desensitization techniques were introduced as healing permitted, alongside thermotherapy to improve local circulation and tissue pliability. Controlled passive and active-assisted mobilization, tendon-gliding exercises and soft tissue stretching were emphasized to restore motion safely. Complementary modalities such as mirror therapy supported cortical reorganization and pain modulation, while bandaging and physical agents (laser, ultrasound, electrotherapy) enhanced edema reduction and tissue repair. Patient education was an essential component, ensuring adherence to prescribed home exercises and recognition of warning signs (e.g., tendon rupture, excessive inflammation).
- Intermediate phase (6–12 weeks): The objectives in this stage were to consolidate early gains, progressively strengthen the hand and improve dexterity and proprioception. Active free mobilization and isometric strengthening exercises were intensified, while neuroproprioceptive facilitation techniques addressed motor control and coordination. Progressive resistance training and stretching helped restore muscle endurance and joint flexibility. Ergotherapy was introduced to retrain fine and gross motor grips in functional contexts, preparing patients for daily activities. Continued use of customized orthoses was recommended when needed, particularly to maintain joint mobility and alignment or to counteract early stiffness. Mirror therapy and desensitization could be maintained for patients with persistent sensory disturbances.
- Advanced functional reintegration phase (≥12 weeks): The final phase was oriented toward restoring complete functional independence and preventing chronic complications such as pain or stiffness. Rehabilitation progressed to complex, task-oriented exercises requiring force, dexterity and coordination. Activities included advanced strengthening, fine motor training and functional tasks such as gripping, throwing and manipulation, adapted to each patient’s professional and personal needs. Functional reintegration also involved targeted programs for athletes or manual workers, ensuring readiness for high-demand activities. Physical modalities (laser, ultrasound, electrotherapy) and thermoformable orthoses were used selectively in cases of persistent pain, deformity, or instability under stress. The ultimate objective was to reestablish maximal function, allowing safe return to occupational and sports performance while preserving quality of life.

**Figure 2:**
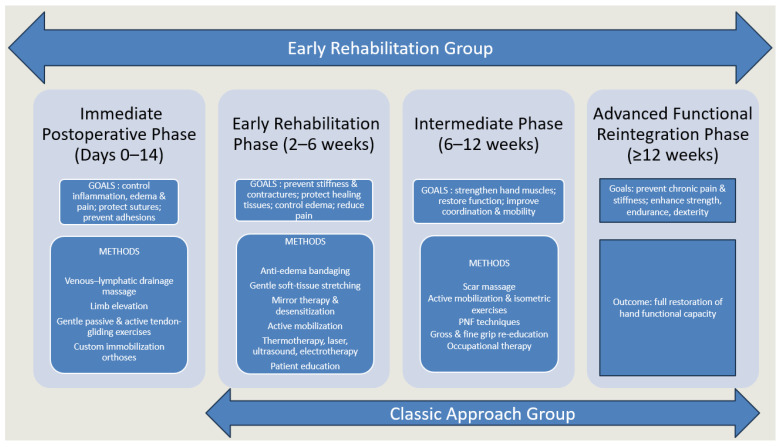
The four progressive phases of the therapeutic program.

The distinction between the two rehabilitation groups lies primarily in the timing of initiation relative to surgery. Patients in the early rehabilitation group followed all four structured phases described above, beginning the immediate postoperative phase shortly after surgical intervention. In contrast, patients in the delayed (classic) rehabilitation group did not undergo the immediate postoperative phase; their rehabilitation started only after removal of the immobilization cast, thus beginning directly with the early, intermediate, and advanced phases (phases 2–4), as shown in Figure 2. Allocation to each group was determined retrospectively, based on the timing of rehabilitation initiation as recorded in the patients’ medical files, which reflected clinical decisions made by the lead surgeons.

### 2.3. Evaluation Protocol

For each patient, a standardized evaluation form was designed, including both objective and subjective parameters relevant to the rehabilitation process. Assessments were conducted at three time points: at the initiation of the rehabilitation program, at four weeks and at twelve weeks after the start of physical therapy. The evaluation protocol is summarized in Table 1.

Active joint mobility was assessed using the Total Active Motion (TAM) score, calculated as the sum of the flexion angles of the metacarpophalangeal, proximal interphalangeal and distal interphalangeal joints, minus the total extension deficit. Measurements were taken bilaterally using the same digital finger goniometer to ensure accuracy and consistency. TAM scores were expressed as percentages, comparing the affected hand to the unaffected one. AROM limitations could be attributed to factors such as fibrosis, adhesions, atrophy, edema or tendon injuries [14,15,16,17].

Grip strength was measured using the Jamar hydraulic hand dynamometer, a tool recognized for its accuracy and reliability. The assessment followed the standardized protocol of the American Society of Hand Therapists (ASHT): patients were seated without arm support, with the elbow flexed at 90° and the arm slightly abducted. Each hand was tested with three maximum-effort contractions, each held for 5 s. The highest value was recorded [18,19,20,21].

Pain intensity was assessed using the Visual Analogue Scale (VAS), a commonly used instrument in clinical research for subjective pain quantification. The scale consists of a 10 cm horizontal line anchored by “no pain” at one end and “worst imaginable pain” at the other. Patients were asked to indicate their pain level over the previous 24 h by marking a point on the line [22,23,24,25,26].

Edema was assessed using the “figure-of-eight” method, a validated and reliable technique for hand circumference measurement. A flexible measuring tape was used, following a standardized path: from the ulnar styloid process across the anterior wrist to the radial styloid, diagonally over the dorsal hand to the metacarpophalangeal joint of the fifth digit, across the palmar side to the metacarpophalangeal joint of the second digit and back diagonally across the dorsum to the starting point [27,28,29].

Hand function was assessed using the QuickDASH questionnaire, a shortened version of the original DASH, validated and widely used in clinical and research settings for musculoskeletal conditions of the upper limb. The tool includes 11 items assessing the patient’s perceived difficulty in performing daily activities such as opening a jar, personal hygiene, household tasks or working without pain. Each item is rated on a 0–5 scale (0 = no difficulty, 5 = severe difficulty) [30,31,32,33,34,35,36].

Patient anxiety levels were evaluated using the GAD-7 (Generalized Anxiety Disorder-7) questionnaire. This validated tool consists of 7 items that assess the frequency of anxiety symptoms experienced over the past two weeks (e.g., restlessness, excessive worry, difficulty relaxing, irritability). The questionnaire takes only a few minutes to complete and enables early identification of anxiety disorders, which are commonly encountered in patients with hand trauma and may significantly impact functional recovery [36,37].

### 2.4. Data Analysis

All data were analyzed using IBM SPSS Statistics 25 and illustrated with Microsoft Office Excel/Word 2025. Quantitative variables were tested for normal distribution using the Shapiro–Wilk Test and were written as averages with standard deviations or medians with interquartile ranges. Quantitative independent variables with normal distribution were tested between groups using Student *t*-Tests (based on the equality of variances observed using Levene’s Test) while quantitative variables with non-parametric distribution were tested between groups using Mann–Whitney U tests. Quantitative variables with normal distribution and repeated measures were tested between measurements using Repeated Measures One-Way ANOVA tests with Greenhouse-Geisser correction (based on the sphericity assumption tested using Mauchly’s test) (with post hoc Bonferroni tests). Quantitative variables with non-parametric distribution and repeated measures were tested between measurements using Friedman tests (with post hoc Dunn-Bonferroni tests). The threshold considered for the significance level for all tests was considered to be α = 0.05.

## 3. Results

To evaluate the impact of rehabilitation timing on clinical outcomes, the study cohort was divided into two main cohorts based on the initiation time of the rehabilitation program. The first group consisted of 26 patients who began rehabilitation after the removal of immobilization, but no later than six weeks post-injury (classic approach group = CAG). The second group included 64 patients who underwent early rehabilitation (early rehabilitation group = ERG), initiated shortly after surgical intervention. Within the early rehabilitation group, two distinct subgroups were identified according to the type of injury: one cohort comprised 21 patients with flexor tendon injuries (either multiple flexor tendons or associated with other injuries–skeletal, nerves or vessels), where the primary objective was to assess the effectiveness of early mobilization in preventing adhesion formation and maintaining flexor tendon gliding and another cohort included 43 patients with other complex hand traumas involving various combinations of skeletal, neural and/or vascular injuries, but without flexor tendons injuries. Although the rehabilitation protocols in this subgroup were more diverse, the early initiation of therapy was consistently applied to evaluate its impact on recovery.

The mean age of the entire cohort was 47.7 ± 14.9 years. Patients in the early rehabilitation group were slightly older (50.2 ± 15.6 years) compared to those in the delayed group (41.7 ± 11.2 years). The study population was predominantly male (86.7%), with a somewhat higher male proportion in the early group (89.1%) than in the delayed group (80.8%). Regarding the mechanism of injury, the combined mechanism (e.g., crush–cut or avulsion–crush injuries) was the most frequent, accounting for 67.8% of all cases. This mechanism was more common in the early group (71.9%) than in the delayed group (57.7%). Sharp (cut) injuries represented 20.0% of cases, followed by crush injuries (7.8%) and avulsion injuries (4.4%). Both crush and avulsion injuries were proportionally more frequent among patients who underwent delayed rehabilitation. In terms of associated structural injuries, vascular injury occurred in 54.4% of all patients, while nerve injury was present in 55.6%. Fractures were documented in 68.9%, flexor tendon injuries in 71.1%, and extensor tendon injuries in 64.4% of the total population. Compared with the early rehabilitation group, the delayed group demonstrated a higher frequency of vascular (65.4% vs. 50.0%), nerve (65.4% vs. 51.6%), and flexor tendon injuries (84.6% vs. 65.6%) (Table 2).

According to the data presented in Table 3, the comparison of score evolution across the entire study group reveals clear trends in functional and clinical recovery over time. Assessments were conducted at three time points: before the initiation of the rehabilitation program (baseline), at 4 weeks (intermediate evaluation), and at 12 weeks (final evaluation). Across all study groups, the data reveal consistent and statistically significant improvement in both clinical and functional outcomes over the course of the rehabilitation program. Assessments at baseline, 4 weeks, and 12 weeks showed clear trends: pain intensity (VAS), edema (EDM), upper limb disability (QuickDASH), and anxiety levels (GAD-7) were highest at baseline and progressively decreased at each subsequent evaluation, indicating effective symptom reduction, while grip strength (GS) and total active motion (TAM) demonstrated steady and significant increases over time, reflecting functional recovery and improved motor performance. Both the overall study group and specific patient cohorts (early rehabilitation group = ERG and classic approach group = CAG) followed this pattern.

Data in Table 4 shows the comparison between the analyzed parameters in their evolution throughout the two groups. Pain intensity decreased from the first measurement towards the second and third measurement, but pain levels were significantly higher in the early approach group than in the classic approach group. Edema measurement decreased from the first measurement towards the second and third measurements, and edema was significantly lower in the early approach group (EAG) than the classic approach group (CAG). Grip strength score evolution from the first measurement towards the second or third measurement was not significantly different between approach groups. Upper limb disability from the first measurement towards the second measurement was not significantly different between approach groups, while the Quick DASH score decreased from the first measurement towards the third measurement. The decrease was significantly higher in the early approach group than the classic approach group. Anxiety decreases from the first measurement towards the second and third measurements and the values were significantly higher in the early approach group than the classic approach group. Total active motion evolution from the first measurement towards the third measurement was not significantly different between the approach groups. The TAM increase from the first measurement towards the third measurement was significantly lower in the early approach group than in the classic approach group, though long-term TAM gains were smaller in the early approach group.

Regarding patients in the subgroup of flexor tendon injuries starting early rehabilitation, data from Table 5 indicate a continuous reduction in pain, edema, disability and anxiety over time. In addition, grip strength and total active motion scores significantly increased from the first to the second and third measurements. Similarly, in patients in the subgroup of other complex hand traumas involving various combinations of skeletal, neural, and/or vascular injuries, starting early rehabilitation, data from Table 5 indicate a continuous reduction in pain, edema, disability, and anxiety over time. In addition, grip strength and total active motion scores significantly increased from the first to the second and third measurements.

Data in Table 6 shows the comparison between the analyzed parameters in their evolution throughout the two subgroups. VAS score evolution from the first measurement towards the second measurement was not significantly different between the 2 injury subgroups, while the VAS decrease from the first measurement towards the third measurement was significantly higher in the other complex injuries subgroup than the flexor tendon injuries subgroup. Edema and the disability evolution from the first measurement to the second and third measurements were not significantly different between the 2 subgroups. The increase in grip strength from the first to the third measurement was not significantly different between subgroups; however, the increase from the first to the second measurement was significantly lower in the other complex injuries subgroup compared to the flexor tendon injuries subgroup. Anxiety changes from the first to the third measurement were not significantly different between subgroups; however, the decrease from the first to the second measurement was significantly lower in the other complex injuries subgroup than in the flexor tendon injuries subgroup. The TAM gain from the first to the second and from the first to the third measurement was significantly lower in the other complex injuries subgroup compared to the flexor tendon injuries subgroup.

## 4. Discussion

### 4.1. Principles of Postoperative Rehabilitation

Hand trauma is considered severe not only because of the potential for permanent physical damage but also due to its impact on a person’s independence, employment, and overall quality of life. The hand is a highly complex anatomical structure responsible for a wide range of essential functions, including fine motor skills, sensory perception, and coordination. Moreover, the psychological effects of hand trauma, such as reduced self-esteem and social isolation, further compound its impact. Given these significant consequences, a timely and accurate diagnosis is crucial to identify the extent of injury and associated complications, such as nerve or tendon damage, infections, or fractures. Appropriate treatment, which may involve surgical intervention, immobilization, and targeted rehabilitation, is essential to maximize functional recovery [16,38,39,40].

Effective rehabilitation after hand trauma depends on the specific structures involved, such as bones, tendons, joints, ligaments, and muscles. Post-surgical complications—like stiffness, adhesions, muscle shortening, and ligament contractures—can severely limit movement if not addressed with a proper therapy plan. The timing of immobilization and the rehabilitation approach depend on the type of surgery performed, as well as ongoing assessment of pain and swelling. Early controlled mobilization techniques are often used to shorten immobilization periods without compromising healing, balancing the protection of healing tissues (e.g., sutures) with the need to prevent secondary complications such as osteoarthritis. The ultimate goal of hand therapy is not only to restore strength and range of motion but also to regain full functional use of the hand for everyday activities [11,41,42].

### 4.2. Overall Outcomes in the Study Group

Throughout our entire study group, regardless of when rehabilitation began, there was a notable decrease over time in edema, pain, anxiety level and upper limb disability. The most significant improvements were observed at the 12-week assessment, indicating not only the effectiveness of the rehabilitation program in addressing physical impairments but also its impact on psychological well-being. Equally important is the progressive enhancement of grip strength and total active motion. The steady gains from week 4 onward reflect the benefits of early mobilization and structured progression, resulting in a tangible restoration of hand function. The improvements marked by week 12 suggest that this period represents a key turning point in the rehabilitation trajectory, where both symptomatic relief and functional gains become most evident. Taken together, these outcomes reinforce the role of comprehensive rehabilitation in facilitating both structural and functional recovery, while also mitigating secondary consequences such as disability and anxiety. The alignment between functional improvements (Quick DASH, grip strength, total active motion) and symptomatic relief (pain, edema, anxiety) underscores the integrative success of the program, supporting its applicability in broader clinical practice.

Consistent with published evidence, objective and patient-reported improvements in hand-trauma rehabilitation exhibit strong concordance, reflecting the integrative success of multimodal programs [43,44,45,46,47].

QuickDASH change thresholds of approximately 12–15 points define minimal clinically important improvement across upper-extremity populations, providing a patient-centered indicator for functional recovery [45,48].

Objective strength and motion correlate with disability scores: grip-strength ratios and absolute grip strength show moderate–strong associations with QuickDASH and PROMIS-UE in hand clinic cohorts, supporting convergent validity between performance and patient-reported outcomes [43,46].

Reliability and clinical utility of motion composites are established, enabling responsive tracking of TAM-linked recovery [49].

Early, controlled mobilization after tendon repair yields superior short-term improvements in motion and function relative to immobilization; however, long-term advantages over passive regimens are not consistently demonstrated, emphasizing the importance of individualized rehabilitation protocols [47,50,51].

Edema-focused interventions (like compression, exercise, selected manual techniques) can reduce subacute hand volume and pain, facilitating motion and activity although the evidence base remains heterogeneous and methodologically limited [52].

Psychological morbidity is common after complex hand trauma. Anxiety, depression and pain interference adversely affect function and targeted screening, while intervention during rehabilitation can optimize overall outcomes, aligning symptomatic relief with functional improvement [39,53].

The cumulative evidence supports the external validity of integrated hand-rehabilitation models, in which concurrent improvements in biomechanical and patient-reported parameters are paralleled by attenuation of pain, edema, and psychological symptoms—a direction likewise approached by the findings of our study.

### 4.3. Classic Approach Group (CAG) Analysis

The analysis of patients who began rehabilitation after immobilization revealed statistically significant improvements across all measured parameters. Pain intensity, edema, disability, and anxiety levels decreased progressively, with clear differences observed between initial, intermediate and final assessments. These results confirm that the therapeutic program effectively addressed both physical symptoms and psycho-emotional impact. In parallel, grip strength and total active motion increased significantly over time, with improvements already evident at the intermediate evaluation and consolidating by week 12. This trajectory highlights the capacity of rehabilitation, even when initiated later, to restore functional mobility and hand strength. Overall, the data demonstrates that the applied protocol contributed substantially to the reduction in pain and edema, improvement of joint mobility and muscle strength, functional recovery and improvement of quality of life in patients with complex post-traumatic hand injuries.

### 4.4. Early Rehabilitation Group (ERG) Analysis

The analysis of patients who began early rehabilitation showed that the application of tendon-gliding techniques, customized thermoformable orthoses and mirror therapy in the immediate postoperative phase, integrated into a comprehensive rehabilitation program, resulted in statistically significant improvements across all measured parameters. Pain intensity, edema, upper limb disability and anxiety levels demonstrated a consistent and significant decrease from baseline to the 4-week and 12-week assessments, with further reductions between the intermediate and final evaluations, confirming progressive clinical improvement. In parallel, grip strength and total active motion exhibited significant increases from the initial measurement to both subsequent evaluations, with additional gains observed between weeks 4 and 12. These results highlight that early rehabilitation not only accelerates recovery of joint mobility and muscle strength but also contributes to functional independence and psychological well-being. The convergence of symptomatic relief and functional enhancement reinforces the validity of early initiation of tendon-gliding exercises, customized orthotic positioning and mirror therapy supporting their role as key components of evidence-based management in complex post-traumatic hand injuries.

Decades ago, early motion emerged as a pivotal concept in the treatment of hand and wrist injuries, particularly fractures of the metacarpals and phalanges. The primary objective was to restore near-normal function by preventing joint stiffness and soft tissue adhesions, which are common complications of prolonged immobilization. However, early mobilization was recognized as a strategy that must be carefully weighed against the risk of compromising fracture stability. The technique was not universally applied but tailored to each patient, initiated only when fracture stability and soft tissue condition permitted. The approach required a multidisciplinary effort, involving specially trained physiotherapists and close clinical and radiologic monitoring by surgeons to ensure both the effectiveness and safety of the rehabilitation process [54,55]. In addition, most studies support early mobilization over delayed rehabilitation in achieving better outcomes following flexor tendon repair. While some research has not demonstrated significant differences between early and delayed mobilization in terms of limb and tendon function, the overall evidence leans toward early movement protocols [56,57,58,59]. The introduction of the Kleinert protocol marked a shift toward early controlled mobilization, and since the 1980s, there has been a growing preference for early active mobilization, supported by experimental and clinical studies. Rehabilitation strategies are typically categorized into immobilization, early controlled mobilization, and early active mobilization, all aiming to improve tendon gliding, prevent complications, and restore function. While early mobilization protocols generally yield better clinical outcomes than immobilization, they vary widely in execution, and no universal gold standard exists. Passive mobilization tends to carry a lower risk of tendon rupture but is often associated with reduced joint mobility compared to early active mobilization [60,61,62,63].

### 4.5. Early Versus Classic Rehabilitation: Comparative Findings

In our study, the comparison between early and late initiation of postoperative rehabilitation in patients with complex hand injuries showed notable differences. While both groups demonstrated significant improvements across the evaluation period, the patterns of recovery suggest distinct advantages and challenges associated with each approach. Patients undergoing early rehabilitation experienced significantly lower edema values, supporting the rationale that prompt initiation of mobilization and therapeutic interventions facilitates faster resolution of swelling. However, this benefit was accompanied by significantly higher pain intensity and anxiety levels compared to patients who began rehabilitation after immobilization was discontinued. These results may reflect the physiological stress and psychological burden associated with engaging the injured hand at an earlier stage of tissue healing, when pain sensitivity and emotional vulnerability are still heightened. In contrast, the late rehabilitation group demonstrated comparatively lower pain and anxiety scores, which may be explained by the longer period of immobilization allowing for partial tissue recovery and psychological adaptation before therapeutic engagement. Nevertheless, this delay in treatment is traditionally associated with risks such as stiffness, functional decline and delayed motor recovery, which must be carefully weighed against the apparent comfort advantages. Taken together, these findings emphasize the complex interplay between physical and psychological outcomes in the timing of rehabilitation. Early rehabilitation may optimize edema control and potentially enhance functional recovery, but clinicians must anticipate and manage the associated increases in pain and anxiety. Late rehabilitation, while less distressing for patients in the early phases, may predispose them to slower functional progress. Functional improvement, as assessed by the QuickDASH, was more pronounced in the early rehabilitation group, particularly by the third evaluation. This finding suggests that initiating therapy soon after surgery, even at the cost of higher early pain and anxiety, facilitates faster recovery of upper limb functionality. The superior functional outcomes in the early group highlight the importance of early mobilization and structured therapeutic interventions in preventing long-term disability and promoting reintegration into daily activities. These results support the notion that functional gains may outweigh the transient discomfort associated with early rehabilitation, reinforcing the clinical relevance of starting rehabilitation protocols without unnecessary delay. Grip strength and total active motion improved significantly over time in both groups, but no significant group differences were observed during the early stages of rehabilitation. This suggests that recovery of muscle strength and joint mobility follows a relatively similar trajectory initially, regardless of the timing of intervention. Although no statistically significant differences were observed between the groups, it is noteworthy that comparable values were reached in the early group considerably earlier than the late group, enabling faster social reintegration. This highlights the importance of carefully balancing early mobilization with adequate protection of healing structures to optimize both short- and long-term outcomes.

### 4.6. Subgroup Analysis: Flexor Tendon vs. Complex Injuries

The subgroup analysis of the patients following an early rehabilitation program revealed that although both categories of patients, those with flexor tendon injuries and those with other complex traumatic injuries, benefited significantly from early rehabilitation, the trajectory and magnitude of recovery differed according to the underlying pathology. Pain intensity decreased consistently in both subgroups. However, long-term reduction (first to the third assessment) was more pronounced in the complex injury group. This may be explained by the fact that patients with multiple tissue injuries, fractures or more extensive trauma initially experience heightened nociception and inflammation, which gradually subsides over time, resulting in a more visible long-term improvement. In contrast, patients with flexor tendon injuries often continue to experience discomfort related to tendon gliding, adhesion or scar sensitivity, factors that may limit the perception of pain relief even under an effective rehabilitation protocol. Edema and functional disability improved significantly and in a comparable manner between groups. This suggests that early mobilization strategies, combined with orthotic positioning, are effective across a broad spectrum of injuries in controlling swelling and supporting the gradual recovery of daily function, regardless of lesion type. The evolution of grip strength showed interesting subgroup dynamics: while long-term improvements were similar, short-term strength gains (first to the second evaluation) were significantly lower in the complex injury group. This finding likely reflects the initial protective response and tissue vulnerability associated with multiple injuries, where pain inhibition, immobilization and tissue healing limit the immediate capacity for force generation. In flexor tendon injuries, however, the structured and progressive gliding protocols allow earlier activation of musculotendinous units, translating into faster early gains in grip strength. Psychological outcomes, as measured by anxiety levels, also followed a differential pattern. Both subgroups reported significant reductions in anxiety levels over time but patients with flexor tendon injuries experienced greater short-term improvements. This may be attributed to the relative predictability of tendon repair outcomes under standardized rehabilitation protocols, which provide patients with a clear therapeutic framework and reassurance. In contrast, patients with complex injuries often face more uncertain recovery trajectories, multiple surgical interventions or visible deformities, all of which may delay psychological adaptation despite eventual functional progress. The most notable divergence was observed in total active motion. Patients with flexor tendon injuries achieved significantly greater gains in finger mobility both at the intermediate and final evaluations compared to those with complex injuries. This highlights the responsiveness of tendon repair cases to well-structured early mobilization protocols, which are specifically designed to optimize tendon gliding, minimize adhesions and preserve joint mobility. In contrast, patients with multiple injuries often face additional barriers such as joint stiffness, soft-tissue scarring or neurovascular involvement, which limit the extent of motion recovery despite rehabilitation efforts.

Taken together, these findings emphasize that early rehabilitation is beneficial across diverse hand trauma profiles, but the magnitude and trajectory of recovery are modulated by injury type. Flexor tendon injuries appear to benefit most in terms of early strength recovery, anxiety reduction and mobility, provided that standardized protocols are strictly followed to balance mobilization with tendon protection. Conversely, complex injury patients, while slower to show strength and mobility gains, report more substantial long-term pain relief, suggesting that rehabilitation strategies in this subgroup should place greater emphasis on long-term functional reintegration and psychosocial support. From a clinical perspective, these results underscore the need for personalized rehabilitation protocols. Flexor tendon repair patients require early, protocol-driven interventions to maximize tendon excursion and functional use, but must be closely monitored for pain, scar adhesions or re-rupture risk. Patients with multiple complex injuries, on the other hand, may benefit from a more gradual progression of mobilization, complemented by interventions targeting pain modulation, edema management and psychological support. The unpredictable postoperative course in flexor tendon injuries further reinforces the importance of multidisciplinary management, involving surgeons, physiotherapists, occupational therapists and psychologists, to adapt protocols dynamically and optimize recovery.

Hand therapy plays a pivotal role in facilitating the return to work for individuals recovering from hand and upper limb conditions, emphasizing the integration of functional rehabilitation with occupational demands. A structured, goal-oriented therapy program that aligns clinical recovery with workplace requirements significantly enhances patients’ confidence, performance, and long-term vocational outcomes [64].

### 4.7. Clinical Implications and Innovative Contributions

This study brings some innovative contributions to the field of postoperative rehabilitation in complex hand trauma. First, the comparative perspective between early and late rehabilitation provides new insights into how the timing of therapeutic intervention influences both physical and psychological recovery trajectories. Previous data in the literature showed that rehabilitation of the injured hand is critical to ensure optimal functional recovery following trauma, as surgical management alone is insufficient without a structured and timely rehabilitation program. Early and properly staged rehabilitation should begin as soon as the surgical repair allows, progressing through gradual phases that balance protection of the healing structures with restoration of mobility, strength and dexterity [65].

While the literature has often emphasized the advantages of early mobilization for tendon healing and joint mobility, few studies have simultaneously addressed its potential limitations in terms of increased pain and anxiety.

By documenting that early rehabilitation led to faster edema resolution and superior functional gains (QuickDASH), but at the cost of higher pain and anxiety levels, this study highlights the clinical challenges that must be considered when designing individualized protocols. Second, the subgroup analysis by injury type represents a novel approach. In particular, differentiating flexor tendon injuries from other complex traumatic lesions allowed the identification of specific recovery patterns. Patients with flexor tendon injuries exhibited greater short-term improvements in grip strength, anxiety reduction and mobility, reflecting the benefits of standardized tendon-gliding protocols. Recent randomized and long-term follow-up studies demonstrated that both passive “place-and-hold” and early active mobilization protocols yield comparable functional recovery and low tendon-rupture rates, confirming the safety and sustained benefits of controlled early motion after flexor tendon repair [59,66].

Conversely, patients with multifocal or complex injuries achieved more substantial long-term pain reduction, underlining the heterogeneous recovery pathways depending on injury typology. Such stratification is rarely presented in rehabilitation studies and adds information for tailoring therapy to specific patient subgroups. Third, the rehabilitation protocol itself incorporated both traditional and advanced interventions in a systematic, stage-based manner. Beyond classical physiotherapy (mobilization, strengthening, scar management), the program integrated custom thermoformable orthoses, manual desensitization, edema control strategies and physical modalities (laser, ultrasound, electrotherapy). Importantly, the inclusion of mirror therapy at early stages is particularly innovative in the context of post-traumatic hand rehabilitation, where its use remains limited compared to neurological populations (e.g., stroke, phantom limb pain). This integration demonstrates the feasibility and potential of applying neurocognitive rehabilitation strategies in orthopedic and trauma settings. Fourth, the study stands out through the systematic inclusion of psycho-emotional evaluation using the GAD-7 scale. While functional outcomes such as grip strength or TAM are routinely assessed, standardized monitoring of anxiety and psychological well-being is far less common in surgical rehabilitation studies. By correlating physical recovery with anxiety dynamics, the study highlights the bidirectional interaction between physical and psychological outcomes, thereby advocating for a more holistic and multidisciplinary rehabilitation framework. Fifth, the multidimensional outcome evaluation is another aspect of novelty. The combined use of objective measures (total active motion, grip strength, edema), subjective pain assessment (VAS), patient-reported functionality (QuickDASH) and psychological screening (GAD-7) provided a comprehensive picture of recovery. This integrative methodology enables clinicians to better understand not only the mechanical restoration of function, but also its impact on daily life activities and emotional health.

The implementation of multiparametric scoring systems, encompassing both objective functional assessments and patient-reported outcome measures, enables a more standardized evaluation of functional outcomes across patients with varying levels of injury complexity. Post-traumatic hand injuries produce a heterogeneous spectrum of functional deficits, and the potential for recovery is influenced by individual patient characteristics as well as by the specific therapeutic strategies employed. Even if the study group included a heterogeneous spectrum of hand injuries involving different anatomical structures and varying degrees of severity, the applied assessment methods (VAS score, edema assessment, grip strength assessment, quick Dash score, GAD-7 score, total active motion assessment) allowed for a standardized quantification of the effectiveness of the implemented rehabilitation protocols. Based on the findings of this study, our ongoing goal is to implement standardized early rehabilitation protocols for complex hand trauma. Naturally, exceptions to this approach remain, including high-severity injuries requiring prolonged immobilization, patients with severe polytrauma or significant systemic compromise, and non-compliant individuals, in whom early rehabilitation is not feasible despite its demonstrated efficacy. Even in patients for whom early rehabilitation is not feasible, a structured and well-supported rehabilitation program initiated as soon as possible provides significant benefits, facilitating the recovery of functional capacity and improving overall quality of life.

### 4.8. Study Limitations

A limitation of the study is the inability to strictly standardize evaluation and treatment protocols in large groups of patients. This challenge arises from the significant variability in post-traumatic hand injuries, which may involve different degrees of complexity, with multiple anatomical structures being affected (osseous, tendinous, neural, vascular, or cutaneous), and which therefore require individualized therapeutic approaches, as opposed to injuries limited to isolated anatomical structures.

We acknowledge that our study cohort is unbalanced, with 64 patients in the early rehabilitation group and 26 in the classic/delayed rehabilitation group. This difference, however, reflects clinical decision-making rather than a methodological limitation. In statistical analysis, comparisons between subgroups with unequal sample sizes were managed by first testing the normality of each subgroup’s data (Shapiro–Wilk test) and equality of variances (Levene’s test). When at least one subgroup showed a non-normal distribution, the non-parametric Mann–Whitney U test was applied, as it is robust to both non-normality and unequal sample sizes. When both subgroups met normality and variance equality assumptions, the independent-samples Student’s *t*-test was used, given its robustness under these conditions even with unequal group sizes. This approach ensured valid and reliable statistical comparisons despite differences in subgroup dimensions. In our practice, early rehabilitation represents the standard approach. Nevertheless, in the case of the 26 patients included in the delayed group, the treating surgeons opted for a more protective strategy involving prolonged immobilization. This decision was based on the severity and complexity of the injuries, where ensuring adequate surgical healing and tissue stability was prioritized before initiating the rehabilitation process. Therefore, the group imbalance resulted from clinical judgment and individualized patient management, not from random or biased sampling. We aimed to reflect real-world clinical practice, where treatment timing is adapted according to injury severity and postoperative stability rather than uniformly applied to all patients.

### 4.9. Future Perspectives

Neuroplasticity is the fundamental mechanism underlying functional recovery after hand trauma. It refers to the nervous system’s intrinsic capacity to reorganize its structural and functional architecture in response to internal and external stimuli, enabling adaptation, learning, and repair. For clinicians involved in hand rehabilitation, a comprehensive understanding of neural plasticity and the capacity of targeted training to modulate cortical reorganization and motor function is essential. Adequate and timely rehabilitation enhances cortical reorganization, optimizes sensorimotor integration, and maximizes the potential for regaining strength, dexterity, and functional independence [67,68,69]. Table 7 summarizes recent and emerging technologies that have been investigated for their capacity to enhance upper limb rehabilitation. These approaches, which include immersive digital platforms, robotic and wearable devices, neuromodulatory techniques, and advanced orthotic solutions, are supported by growing experimental and clinical evidence. Importantly, they hold translational relevance for post-traumatic hand recovery, where neuroplasticity-driven interventions may optimize functional restoration and long-term outcomes.

## 5. Conclusions

This study highlights the overall effectiveness of structured postoperative rehabilitation for complex hand trauma, regardless of whether therapy is initiated early or after a period of immobilization. Early rehabilitation, though associated with increased initial pain and anxiety, leads to faster improvements in edema reduction, grip strength, joint mobility, and upper limb function. These benefits support its role in preventing long-term disability and enhancing functional recovery, provided that pain and psychological stress are proactively managed. In contrast, delayed rehabilitation appears to be less distressing in the early stages but may result in slower functional progress, emphasizing the importance of timing in therapeutic decision-making. Recovery trajectories also varied based on injury type, with patients recovering from flexor tendon injuries showing quicker early gains, likely due to the structured nature of tendon-specific rehabilitation protocols. Those with more complex or multifocal injuries, while slower to improve initially, experienced more pronounced long-term pain relief, indicating different therapeutic needs based on injury complexity. By highlighting the interplay between timing, injury typology, physical interventions, and psycho-emotional dimensions, while also addressing the challenge of heterogeneous trauma populations, the findings suggest that the future of hand trauma rehabilitation lies in personalized, multimodal, and multidisciplinary approaches.

## Figures and Tables

**Table 1:** Evaluation protocol and assessment tools during the rehabilitation process.

Parameter Evaluated	Assessment Tool/Method	Details
1. Active Range of Motion (AROM)	Total Active Motion (TAM) Score	Sum of MCP, PIP, and DIP flexion minus total extension deficit; bilateral measurement with digital goniometer.
2. Grip Strength (GS)	Jamar Hydraulic Hand Dynamometer	Standard ASHT protocol; three maximal 5 s contractions per hand; highest value recorded.
3. Pain Intensity	Visual Analogue Scale (VAS)	10 cm horizontal line from “no pain” to “worst imaginable pain”; patient marked pain level from last 24 h.
4. Edema (EDM)	Figure-of-Eight Measurement	Standardized tape measurement route around hand joints; reliable for hand swelling assessment.
5. Hand Function	QuickDASH Questionnaire (QD)	11 items rated 0–5; assesses difficulty in daily activities and functional limitations.
6. Anxiety Level	Generalized Anxiety Disorder Questionnaire (GAD-7)	7 items evaluating anxiety symptoms in the past 2 weeks; facilitates early detection of psychological impact.

**Table 2:** Characteristics of the study cohorts.

	Total	ERG	CAG
Mean Age (years) ± SD	47.7 ± 14.9	50.2 ± 15.6	41.7 ± 11.2
Male	86.70%	89.10%	80.80%
Female	13.30%	10.90%	19.20%
Sharp injury	20.00%	21.90%	15.40%
Crush injury	7.80%	4.70%	15.40%
Avulsion	4.40%	1.60%	11.50%
Combined mechanism	67.80%	71.90%	57.70%
Vascular injury	54.40%	50.00%	65.40%
Nerve injury	55.60%	51.60%	65.40%
Fracture	68.90%	68.80%	69.20%
Flexor tendon injury	71.10%	65.60%	84.60%
Extensor tendon injury	64.40%	64.10%	65.40%

CAG = classic approach group, ERG = early rehabilitation group.

**Table 3:** Temporal changes in the analyzed parameters across study groups throughout rehabilitation.

Parameter	Group	Baseline (Mean ± SD)	4 Weeks (Mean ± SD)	12 Weeks (Mean ± SD)	*p*-Value
VAS	Total	8.91 ± 1.85	5.24 ± 1.98	2.13 ± 1.76	<0.001
	CAG	7.46 ± 2.64	5.15 ± 2.47	3.08 ± 2.10	
	ERG	9.50 ± 0.94	5.28 ± 1.76	1.75 ± 1.45	
EDM	Total	61.48 ± 7.18	60.23 ± 6.98	59.09 ± 6.76	<0.001
	CAG	61.31 ± 8.05	59.58 ± 7.58	58.23 ± 7.24	
	ERG	61.55 ± 6.87	60.50 ± 6.76	59.44 ± 6.59	
GS	Total	1.06 ± 3.15	3.77 ± 5.23	13.14 ± 8.69	<0.001
	CAG	2.52 ± 5.43	6.57 ± 7.94	14.69 ± 13.28	
	ERG	0.47 ± 1.05	2.64 ± 3.01	12.51 ± 5.95	
QD	Total	85.08 ± 10.88	71.65 ± 18.18	35.54 ± 24.60	<0.001
	CAG	82.10 ± 13.28	67.82 ± 23.92	47.98 ± 28.84	
	ERG	86.24 ± 9.66	73.14 ± 15.36	30.68 ± 21.03	
GAD-7	Total	18.31 ± 4.05	14.32 ± 5.64	7.92 ± 6.59	<0.001
	CAG	17.31 ± 5.75	14.65 ± 6.02	10.04 ± 6.00	
	ERG	18.72 ± 3.07	14.19 ± 5.53	7.06 ± 6.67	
TAM	Total	35.92 ± 26.63	49.08 ± 26.55	70.51 ± 24.22	<0.001
	CAG	21.70 ± 24.58	39.22 ± 28.80	58.13 ± 29.18	
	ERG	41.70 ± 25.39	53.08 ± 24.70	75.53 ± 20.05	

VAS = pain intensity, EDM = edema, GS = grip strength, QD = quick Dash, upper limb disability, GAD-7 = anxiety levels, TAM = total active motion, CAG = classic approach group, ERG = early rehabilitation group.

**Table 4:** Comparison between the two different approach groups (classic/early) in relation to the analyzed parameters.

Group/Dif VAS I-II	Mean ± SD	*p*
CAG (*p* = 0.151)	2.3 ± 1.43	<0.001
ETG (*p* = 0.001)	4.21 ± 1.71	
**Group/Dif VAS I-III**	**Mean ± SD**	***p***
CAG (*p* = 0.090)	4.38 ± 2.11	<0.001
ERG (*p* = 0.003)	7.75 ± 1.35	
**Group/Dif EDM I-II**	**Mean ± SD**	***p***
Classic (*p* = 0.010)	1.73 ± 1.18	0.004
Early (*p* < 0.001)	1.05 ± 0.51	
**Group/Dif EDM I-III**	**Mean ± SD**	***p***
CAG (*p* = 0.166)	3.08 ± 1.81	0.007
ERG (*p* < 0.001)	2.11 ± 1	
**Group/Dif GS I-II**	**Mean ± SD**	***p***
CAG (*p* = 0.001)	−4.06 ± 4.3	0.061
ERG (*p* < 0.001)	−2.17 ± 2.5	
**Group/Dif GS I-III**	**Mean ± SD**	***p***
CAG (*p* = 0.025)	−12.17 ± 10.9	0.487
ERG (*p* = 0.436)	−12 ± 5.55	
**Group/Dif QD I-II**	**Mean ± SD**	***p***
CAG (*p* = 0.001)	14.38 ± 13.4	0.456
ERG (*p* < 0.001)	13.1 ± 10	
**Group/Dif QD I-III**	**Mean ± SD**	***p***
CAG (*p* = 0.001)	34.11 ± 22.2	<0.001
ERG (*p* = 0.001)	55.56 ± 17	
**Group/Dif GAD-7 I-II**	**Mean ± SD**	***p***
CAG (*p* = 0.001)	2.65 ± 2.92	0.011
ERG (*p* = 0.003)	4.53 ± 3.18	
**Group/Dif GAD-7 I-III**	**Mean ± SD**	***p***
CAG (*p* = 0.367)	7.27 ± 4.73	<0.001
ERG (*p* < 0.001)	11.66 ± 5.21	
**Group/Dif TAM I-II**	**Mean ± SD**	***p***
CAG (*p* = 0.174)	−17.53 ± 11.98	0.016
ERG (*p* < 0.001)	−11.38 ± 8.66	
**Group/Dif TAM I-III**	**Mean ± SD**	***p***
CAG (*p* = 0.048)	−36.43 ± 22.19	0.862
ERG (*p* = 0.002)	−33.84 ± 16.16	

I = measurement at the beginning of the rehabilitation program, II = measurement at 4 weeks, III = measurement at 12 weeks after the beginning of the rehabilitation program; VAS = pain intensity, EDM = edema, GS = grip strength, QD = quick Dash, upper limb disability, GAD-7 = anxiety levels, TAM = total active motion, CAG = classic approach group, ERG = early rehabilitation group.

**Table 5:** Evolution score comparison of the analyzed parameters in the flexor tendon injuries subgroup and the other complex hand injuries subgroup.

Parameter	Time Point	Flexor Tendon Injuries (Mean ± SD)	Other Complex Hand Injuries (Mean ± SD)	*p*-Value
VAS	Baseline	9.05 ± 1.2	9.72 ± 0.7	<0.001
	4 weeks	5.38 ± 1.62	5.23 ± 1.85	
	12 weeks	2 ± 1.58	1.63 ± 1.39	
EDM	Baseline	58.48 ± 7	63.05 ± 6.36	<0.001
	4 weeks	57.43 ± 6.87	62 ± 6.25	
	12 weeks	56.43 ± 6.78	60.91 ± 6.04	
GS	Baseline	0.33 ± 0.79	0.53 ± 1.16	<0.001
	4 weeks	3.14 ± 2.74	2.39 ± 3.14	
	12 weeks	12.33 ± 5.52	12.6 ± 6.21	
QD	Baseline	81.65 ± 11.75	88.48 ± 7.65	<0.001
	4 weeks	67.6 ± 14.52	75.85 ± 15.18	
	12 weeks	27.69 ± 19.8	32.15 ± 21.68	
GAD-7	Baseline	16.71 ± 3.6	19.7 ± 2.24	<0.001
	4 weeks	10.57 ± 5.46	15.95 ± 4.68	
	12 weeks	4.38 ± 5.39	8.37 ± 6.89	
TAM	Baseline	35.81 ± 26	44.57 ± 24.85	<0.001
	4 weeks	52.13 ± 24.9	53.54 ± 24.88	
	12 weeks	77.6 ± 20	74.51 ± 20.2	

VAS = pain intensity, EDM = edema, GS = grip strength, QD = quick Dash, upper limb disability, GAD-7 = anxiety levels, TAM = total active motion.

**Table 6:** Comparison between the two subgroups (flexor injury/other complex hand injury) in relation to the analyzed parameters.

Injury/Dif VAS I-II	Mean ± SD	*p*
Flexor (*p* = 0.029)	3.67 ± 1.19	0.136
Other (*p* = 0.012)	4.49 ± 1.87	
**Injury/Dif VAS I-III**	**Mean ± SD**	***p***
Flexor (*p* = 0.053)	7.05 ± 1.07	0.004
Other (*p* = 0.006)	8.09 ± 1.36	
**Injury/Dif EDM I-II**	**Mean ± SD**	***p***
Flexor (*p* < 0.001)	1.05 ± 0.49	1.000
Other (*p* < 0.001)	1.05 ± 0.53	
**Injury/Dif EDM I-III**	**Mean ± SD**	***p***
Flexor (*p* = 0.064)	2.05 ± 1.2	0.668
Other (*p* < 0.001)	2.14 ± 0.89	
**Injury/Dif GS I-II**	**Mean ± SD**	***p***
Flexor (*p* = 0.020)	−2.81 ± 2.4	0.039
Other (*p* < 0.001)	−1.86 ± 2.51	
**Injury/Dif GS I-III**	**Mean ± SD**	***p***
Flexor (*p* = 0.458)	−12 ± 5.45	0.070
Other (*p* = 0.438)	−12.07 ± 5.65	
**Injury/Dif QD I-II**	**Mean ± SD**	***p***
Flexor (*p* = 0.015)	14.07 ± 8.14	0.246
Other (*p* < 0.001)	12.63 ± 10.95	
**Injury/Dif QD I-III**	**Mean ± SD**	***p***
Flexor (*p* = 0.985)	53.96 ± 13.65	0.232
Other (*p* < 0.001)	56.33 ± 18.52	
**Injury/Dif GAD-7 I-II**	**Mean ± SD**	***p***
Flexor (*p* = 0.460)	6.14 ± 2.79	0.006
Other (*p* = 0.003)	3.74 ± 3.08	
**Injury/Dif GAD-7 I-III**	**Mean ± SD**	***p***
Flexor (*p* = 0.005)	12.33 ± 3.83	0.874
Other (*p* < 0.001)	11.33 ± 5.78	
**Injury/Dif TAM I-II**	**Mean ± SD**	***p***
Flexor (*p* = 0.020)	−16.32 ± 10.8	0.003
Other (*p* = 0.016)	−8.97 ± 6.23	
**Injury/Dif TAM I-III**	**Mean ± SD**	***p***
Flexor (*p* = 0.143)	−41.82 ± 16.28	0.004
Other (*p* = 0.002)	−29.94 ± 14.77	

I = measurement at the beginning of the rehabilitation program, II = measurement at 4 weeks, III = measurement at 12 weeks after the beginning of the rehabilitation program, VAS = pain intensity, EDM = edema, GS = grip strength, QD = quick Dash, upper limb disability, GAD-7 = anxiety levels, TAM = total active motion.

**Table 7:** Recent and emerging technologies used in upper limb rehabilitation [70,71,72,73,74,75,76,77,78,79,80,81,82,83,84,85,86].

Technology Type	Working Mode/Functionality Addressed	Advantages	Limitations/Challenges
Virtual/Augmented Reality (VR/AR)	Immersive, task-specific environments; real-time visual and kinematic feedback; gamified training.	Enhances engagement and motivation; enables high-repetition practice; supports pain modulation (phantom pain, CRPS).	Requires equipment and technical support; risk of cybersickness; transfer to daily activities still variable.
Robotics	End-effector systems guiding hand/arm movement; repetitive assist-as-needed training.	Increases therapy volume; ensures consistent dosing; provides objective data on performance.	High cost; bulky devices; limited availability in smaller centers; ADL transfer not always demonstrated.
Exoskeletons (Soft/Hard)	Wearable devices assisting or resisting finger/wrist motion.	Portable (especially soft devices); adaptable assistance; potential for home-based use.	Comfort, durability, and cost remain barriers; clinical evidence still limited.
Surface EMG (sEMG) Biofeedback	Records muscle activity; provides visual/game feedback; trains selective recruitment and control.	Improves voluntary activation; supports cortical reorganization; feasible for home-based training.	Requires patient understanding and calibration; may be less effective in cases with very weak or absent signals.
Brief Therapeutic Electrical Stimulation (TES)	Short intra-/post-operative nerve stimulation to accelerate regeneration.	Enhances axonal regrowth; improves motor/sensory recovery after nerve repair; only one session needed.	Currently experimental in many centers; requires surgical integration; long-term clinical data still emerging.
Orthoses (3D-printed, dynamic)	Custom immobilization or guided motion; scan-to-fit printing.	Lightweight, ventilated, higher comfort/compliance; customizable with hinges/sensors.	Access to 3D printing technology; reprinting needed for swelling changes; not yet universally available.
Telerehabilitation/Remote rehabilitation	Remote supervision via video, digital exercise platforms, and sensors.	Expands access; maintains adherence; outcomes comparable to in-person in selected conditions. Exercise program can be performed on a touchscreen tablet-based app in combination with face-to-face physiotherapy	Limited for complex cases needing hands-on therapy; digital literacy and internet access required.
Sensory-Motor Re-education	Tactile discrimination, mirror therapy, graded motor imagery.	Improves sensory recovery after nerve trauma; addresses cortical remapping; helpful for phantom pain.	Time-intensive; variable efficacy across studies; requires high patient engagement.

## Data Availability

The raw data supporting the conclusions of this article will be made available by the authors upon request.
